# High prevalence of low faecal elastase levels in individuals with type 1 diabetes is associated with diabetes duration but not with marked alterations in food intake or clinical symptoms

**DOI:** 10.1007/s00125-026-06829-9

**Published:** 2026-08-08

**Authors:** Douwe F. de Wit, Coco M. Fuhri Snethlage, Johannes H. M. Levels, Elena Rampanelli, Abraham S. Meijnikman, Max Nieuwdorp, Nordin M. J. Hanssen

**Affiliations:** 1 Department of Experimental Vascular Medicine, Amsterdam UMC, Amsterdam, the Netherlands; 2 The Netherlands Diabeter Centrum Amsterdam, Amsterdam, the Netherlands; 3 Amsterdam Gastroenterology Endocrinology Metabolism (AGEM), Amsterdam, the Netherlands; 4 Amsterdam Institute for Immunology and Infectious Diseases, Amsterdam, the Netherlands

**Keywords:** Clinical science, Epidemiology, Gastro-entero pancreatic factors

## Abstract

**Aims/hypothesis:**

Although exocrine pancreatic insufficiency (EPI) is thought to be common in type 1 diabetes, little is known about the relationship of EPI with type 1 diabetes parameters or food intake and abdominal complaints. Characterising individuals with EPI could help prevent EPI-related complications such as malabsorption and osteoporosis.

**Methods:**

In a cross-sectional cohort of 443 individuals with type 1 diabetes (62% female, median age 42 years [IQR 29–54], mean BMI 25±4 kg/m^2^, median diabetes duration 16 years [IQR 6–29]), we associated faecal elastase (FE) levels, as a marker of EPI, with type 1 diabetes parameters and clinical features, using linear and Poisson regression models.

**Results:**

The mean FE level (±SD) was 349.5±146.8 µg/g faeces. A number of variables were independently associated with lower FE levels: older age (−10.97 µg/g faeces per 10 years; 95% CI −21.02, −0.92), longer diabetes duration (−13.09 µg/g per 10 years; 95% CI −23.54, −2.64), higher HbA_1c_ (−1.44 mmol/mol per 1 SD increase in FE levels, 95% CI −2.59, −0.30), male sex (−54.07 µg/g; 95% CI −81.41, −26.73) and smoking (−85.06 µg/g; 95% CI −128.52, −41.60). In a linear analysis, we found no evidence of associations between FE levels and markers of residual beta cell function, liver disease, macronutrient intakes or gastrointestinal complaints. Using a cut-off of 200 µg/g faeces, 76 individuals (17%) were found to have EPI, which was associated with a 4.31 mmol/mol (0.39%) higher HbA_1c_ (95% CI 1.35, 7.26), an 8% higher mean glucose (95% CI 2, 14), a 9% lower fibre intake (95% CI 1, 17) and an 8% lower energy intake (95% CI 1, 15).

**Conclusions/interpretation:**

The lack of evidence for associations with clinical parameters in this relatively healthy population of individuals with type 1 diabetes does not support the use of FE for routine screening for EPI. However, the high prevalence of low FE levels and their association with lower food intake and higher glucose levels justify a need for clinical awareness for EPI and its complications, especially in individuals with long diabetes duration.

**Graphical Abstract:**

**Figure:**
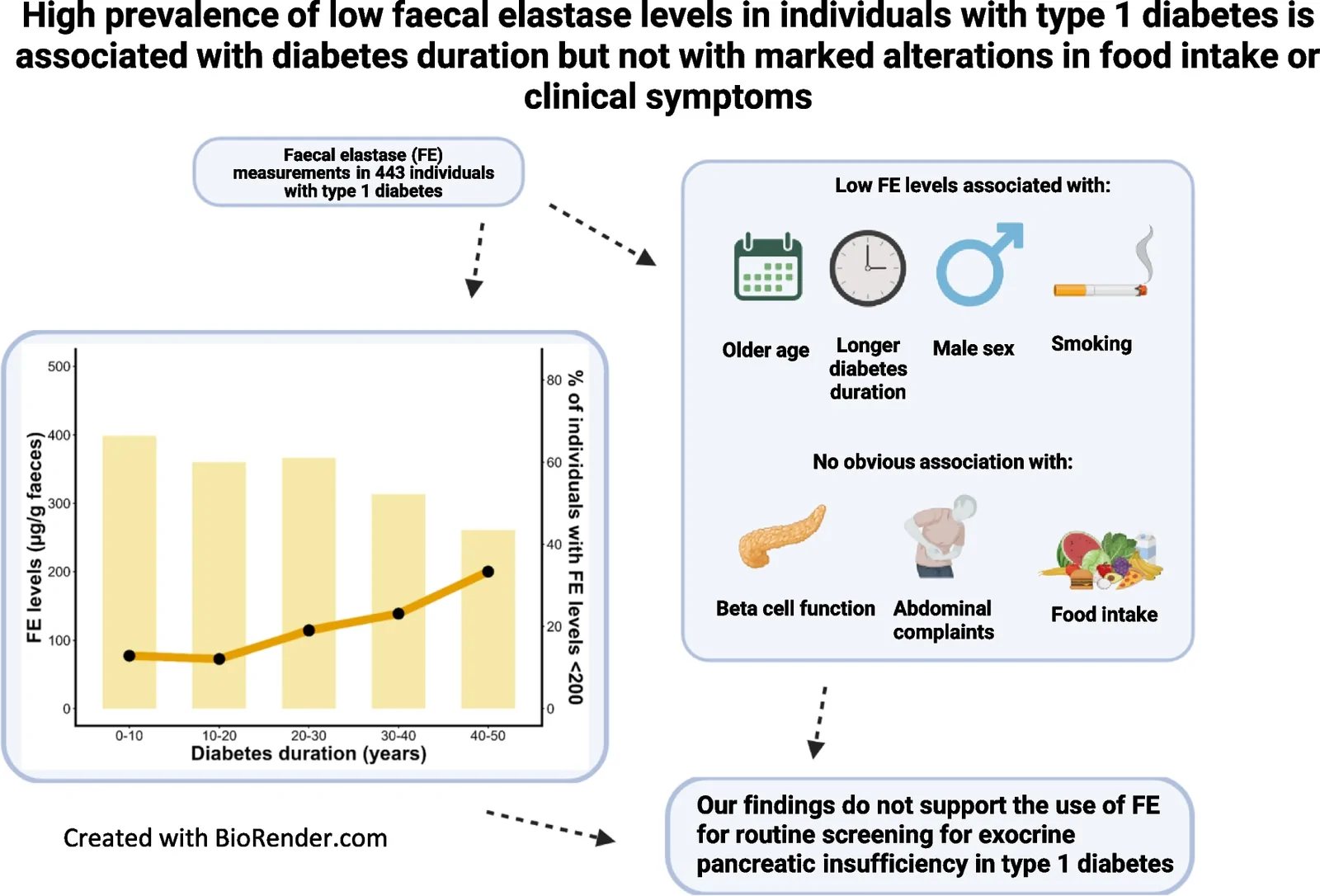

**Supplementary Information:**

The online version of this article (https://doi.org/10.1007/s00125-026-06829-9) contains peer-reviewed but unedited supplementary material.

**Figure:**
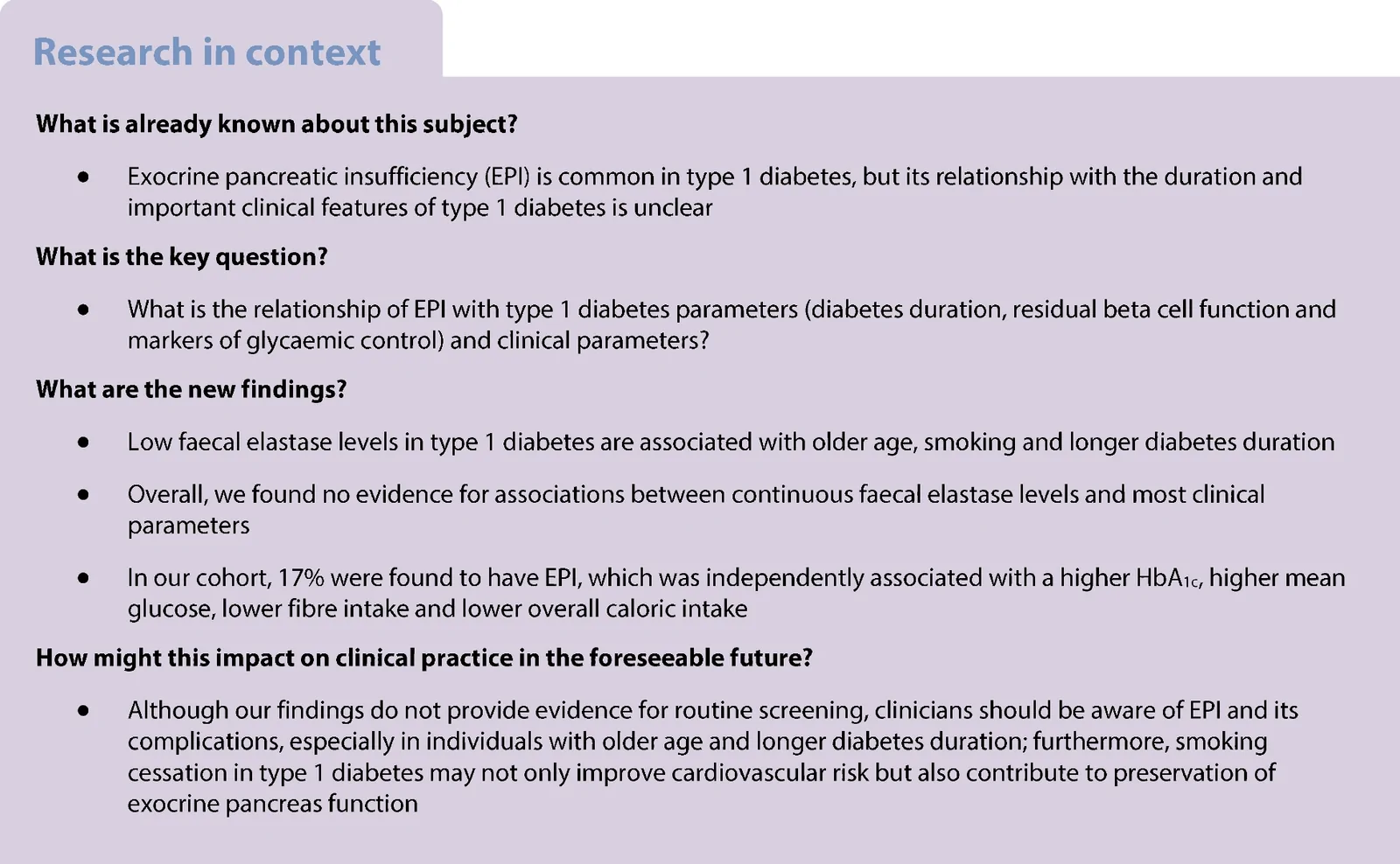

## Introduction

Although type 1 diabetes is believed to be mainly driven by T cell-mediated beta cell destruction, leading to life-threatening insulin deficiency, abnormalities of the exocrine pancreas and exocrine pancreatic insufficiency (EPI) have also been reported in type 1 diabetes [1]. However, the link between EPI and beta cell function and/or glycaemic control has not been elucidated in type 1 diabetes. The first line of evidence of EPI in type 1 diabetes comes from observations that the pancreatic volume is decreased by 20–50% in children and adults with type 1 diabetes [2], and the decrease in pancreatic weight appears to precede the diagnosis of type 1 diabetes [3]. Given that only 1–2% of the pancreatic mass consists of islets, the reduced pancreas size necessarily involves the exocrine compartment. Moreover, numerous studies have described that the exocrine pancreatic compartment in type 1 diabetes is characterised by reduced acinar cell proliferation (possibly due to insulinopenia [4]), cross-reacting antibodies [5], acinar immune cell infiltration [6] and complement deposition [7], emphasising the endocrine–exocrine entanglement. To diagnose EPI, direct functional tests, such as faecal fat quantification or stimulation tests using an nasoduodenal catheter, are considered to be the most accurate, but these are expensive, time-consuming and often not available [8]. Measurement of faecal elastase (FE) levels is a frequently used indirect diagnostic test for EPI, with a value of <200 µg/g faeces considered abnormal in international guidelines [9]. Although low FE levels are often reported in individuals with type 1 diabetes (FE levels <200 µg/g faeces in up to 50% of individuals [10–12]), how EPI and beta cell dysfunction affect each other remains unclear. In addition, well-known complications of EPI such as osteoporosis and malabsorption are more prevalent in individuals with type 1 diabetes [13–15]. However, the true prevalence of EPI in type 1 diabetes is unknown [9]. To address this, we used a cohort of 500 type 1 diabetes individuals and investigated the association between EPI and type 1 diabetes parameters (diabetes duration, residual beta cell function and markers of glycaemic control), as well as the prevalence of liver disease and abdominal complaints and a potential link to macronutrient intake. Characterising the prevalence and clinical features of EPI in type 1 diabetes is important, because screening for complications associated with EPI, such as malabsorption and osteoporosis, may be necessary in these individuals.

## Methods

### Study population

The GUTDM1 cohort is a cross-sectional, observational cohort comprising 500 individuals with type 1 diabetes who were recruited within the Amsterdam region from 2020 to 2022 as described in detail previously [16, 17]. Participants were primarily recruited from outpatient clinics in hospitals in the Amsterdam region. No data are available on the characteristics of the source population. Potential participants were either contacted by phone by the investigators or asked to participate by their internist or diabetes nurse. Participants had to be >18 years of age to participate, and the type 1 diabetes diagnosis had to have been made by a clinician. In accordance with usual care, individuals with type 1 diabetes received education on medical nutritional therapy and carbohydrate counting from a dedicated type 1 diabetes dietitian. We included individuals with type 1 diabetes regardless of diabetes duration, sex, ethnicity, socioeconomic factors, device use, and the presence of micro- or macrovascular complications or gastrointestinal conditions such as coeliac disease. Exclusion criteria were active infection at time of inclusion, unwillingness to donate faeces, urine and/or blood, inability to provide informed consent, or absence of the large bowel (i.e. colostomy). Participant sex was self-reported. We did not collect data on race/ethnicity.

### Data collection

The study consisted of a single hospital visit, at which the medical history was reported, a physical examination was performed, and fasting blood was drawn. The day before the hospital visit, participants collected a fresh stool sample and filled in standardised questionnaires on their insulin use, pump use and use of real-time continuous glucose monitoring (CGM) devices or intermittently scanned CGM devices, as well as abdominal complaints (using the validated Gastrointestinal Symptom Rating Scale [GSRS] [18, 19]), exercise (hours per week), alcohol consumption (units per week) and smoking status. The GSRS consists of 15 questions on abdominal complaints, with scores ranging from 1 (no complaints) to 7 (very severe complaints). The GSRS was divided into five subdomains (abdominal pain, gastro-oesophageal reflux disease, constipation, indigestion and diarrhoea), each containing 2–4 questions as described previously [18]. A mean score of ≥3 for each subdomain was considered as positive for each condition. For 3 days before the visit (two weekdays and one weekend day), participants recorded data on macronutrient intake in an online food diary, using the validated web-based application ‘Eetmeter’, version 5.1.5, created by the Netherlands Nutrition Centre, a Dutch governmental institution on healthy nutrition (eetmeter.voedingscentrum.nl). An extensive description of the methodology for macronutrient data collection and CGM metrics has been published previously [17]. We measured the stimulated urinary C-peptide to creatinine ratio (UCPCR, nmol/mmol) and proinsulin as previously described [16]. The fibrosis score was assessed by liver stiffness measurement using vibration-controlled transient elastography (LSM-VCTE) using FibroScan 530 Compact (Echosens, France).

### Faecal elastase measurement

The FE measurements were performed on 100 mg of the fresh stool sample that individuals collected (stored at −70°C in our laboratory), using a validated ELISA with polyclonal antibodies (product code BS-86-01-SK15, Bioserv Diagnostics [20]) at the Experimental Vascular Medicine Laboratory at Amsterdam UMC, Amsterdam. The lower and upper detection limits are 15 and 500 µg/g faeces, respectively, and the within-run and between-run coefficients of variance are 5% and 6.3%, respectively. Individuals with watery stool samples or self-reported watery stools in the week before collection (recorded in the GSRS as ‘severe discomfort’ or ‘very severe discomfort’ due to loose stools) were excluded from analysis due to the risk of underestimation of FE levels.

### Ethical considerations

The ethical principles of the Declaration of Helsinki were followed, and the study was conducted in accordance with the Dutch Medical Research Involving Human Subjects Act (WMO). All included participants signed an informed consent form. This study was approved by the medical-ethical committee of Amsterdam UMC (Dutch Central Commission of Human Research number NL73189.018.20).

### Statistical analysis

Baseline characteristics are presented as means ± SD or medians (IQR), as appropriate for continuous variables, and as percentages for categorical variables. The data distribution of baseline variables was checked using histograms. Non-normally distributed outcomes (macronutrient intakes, GSRS score, liver fibrosis score, fasting glucose and mean glucose) were log_e_-transformed to obtain a normal distribution, or, in the case of CGM metrics, dichotomised according to international guidelines [21]: time in range (TIR) < 70% or ≥70%, time below range (TBR) ≥ 4% or <4%, time above range (TAR) ≥ 25% or <25%, glucose variability (GCV) ≥ 36% or <36%. Fasting C-peptide and UCPCR were also dichotomised according to the levels measured: fasting C-peptide: >0.05 or ≥0.05 nmol/l and UCPCR ≥0.01 or <0.01 nmol/mmol. FE levels were converted to *z* scores to express the association according to the SD of FE levels (1 SD = 147 µg/g faeces). Baseline characteristics of individuals included in the analysis versus individuals who were not included (electronic supplementary material [ESM] Table 5) were compared using two-sided unpaired *t* tests or Mann–Whitney *U* tests. To establish the associations between age, diabetes, duration, sex, BMI, smoking, alcohol use and FE levels, we generated a linear regression model with FE levels as the dependent variable and the other variables as independent variables. To establish the associations of FE levels with macronutrient intakes, we used a linear regression model with macronutrient intakes as outcomes, representing relative differences in intakes per 1 SD (147 µg/g) increase in FE. The crude ratios and adjusted ratios are presented (model 1: adjusted for age, sex, diabetes duration, BMI, smoking and alcohol use; model 2: adjustments as for model 1 + total energy intake). PP plots and QQ plots of the residuals were checked for normality to meet the linear model assumptions. For the linear regressions, outcomes are expressed as B (unstandardised regression coefficient), expressing the change in unit outcome per 1 SD increase in FE levels for non-transformed data, or exp(B) for log_e_-transformed data, expressing the fold change per 1 SD increase in FE levels. To assess the association between FE levels and the dichotomised outcomes, we performed Poisson regression with robust standard errors to estimate prevalence ratios and corresponding 95% CI intervals. As a sensitivity analysis, we dichotomised FE levels to <200 µg/g faeces (EPI group) or ≥200 µg/g faeces (non-EPI group), in line with international guidelines [9]. The same models were used for these analyses. For the nutritional data, extreme outliers were excluded from the analyses by erasing the first and 100th percentile of total energy intake (*n*=10), as described previously [22]. For all analyses, a *p* value <0.05 was considered statistically significant. RStudio version 2022.02.3 was used for all statistical analyses (R Foundation for Statistical Computing, Vienna, Austria).

## Results

Of the 500 individuals in the GUTDM1 cohort, faecal material for FE measurement was available for 452 individuals. We excluded nine individuals with self-reported watery stools to prevent false-positive classification of EPI, meaning that 443 individuals were suitable for analysis. The mean FE level (±SD) was 349.5±146.8 µg/g faeces. Seventy-six individuals (17%) had EPI, and 367 (83%) did not. Table 1 summarises the baseline characteristics for the participants. We observed no major violation of the normal distribution for the residuals of the adjusted models (see ESM Figs 1 and 2).

**Table 1:** Baseline characteristics of study participants

Characteristic	
Total number of participants	443
Female	276 (62.3)
Age, years	42.00 (29.00, 54.00)
BMI, kg/m^2^	25.36±4.19
Diabetes duration, years	16.00 (6.00, 29.00)
Insulin use, U/day	37.08 (26.96, 53.00)
Insulin use, U kg^−1^ day^−1^	0.53±0.29
HbA_1c_, mmol/mol	54.79±11.70
HbA_1c_,%	7.16±1.07
Fasting glucose, mmol/l	9.04±3.49
Mean glucose, mmol/l	8.90±2.00
FE, µg/g faeces	349.5±146.8
Haemoglobin, mmol/l	8.68±0.86
Thrombocytes, 10^9^/l	258.64±58.43
Leukocytes, 10^9^/l	5.60 (4.60, 6.80)
eGFR, ml/min per 1.73 m^2^^a^	100.63±18.45
Alkaline phosphatase, IU/l	77.26±22.19
γ-glutamyl transferase, U/l	15.00 (11.00, 21.00)
Bilirubin, µmol/l	10.63±5.90
ASAT, IU/l	23.00 (19.00, 28.00)
ALAT, IU/l	20.00 (16.00, 27.00)
Fibrosis score, kPa^b^	4.70±1.55
CRP, mg/l	1.20 (0.60, 2.35)
Fasting C-peptide, nmol/l	0.05 (0.05, 0.08)
UCPCR, nmol/mmol	0.00 (0.00, 0.41)
UCPCR secretors	220 (49.7)
Total cholesterol, mmol/l	4.71±0.90
HDL-cholesterol, mmol/l	1.79±0.45
Non-HDL-cholesterol, mmol/l	2.91±0.82
LDL-cholesterol, mmol/l	2.58±0.78
Triglycerides, mmol/l	0.61 (0.45, 0.84)
Smoking	43 (9.7)
Alcohol intake, units/week	2.00 (0.00, 6.00)
GADA	1.60 (1.00, 46.75)
IA-2A	5.00 (5.00, 38.00)
TIR,%	67.00 (52.00, 80.50)
TAR,%	29.00 (16.00, 45.00)
TBR,%	2.00 (1.00, 4.00)
Glucose variability,%	33.80 (28.60, 39.27)
Fibre intake, g/day	20.21±9.16
Carbohydrate intake, g/day	160.24±62.05
Fat intake, g/day	74.48±30.93
Protein intake, g/day	69.66±24.20
Caloric intake, kJ/day	6821.34±1972.63
Sugar intake, g/day	61.84±31.30
Fat intake, % of total caloric intake	40.55±9.37
GSRS score, total	25.67 (20.00, 34.00)
Symptoms	
Diarrhoea	52 (11.7)
Abdominal pain	96 (21.7)
GORD	114 (25.7)
Indigestion	31 (7.0)
Constipation	64 (14.4)

Values for categorical variables are *n* (%); values for continuous variables are means ± SD or median (Q1, Q3)

^a^Measured using the CKD-EPI method

^b^Assessed by liver stiffness measurement using vibration-controlled transient elastography (LSM-VCTE)

ALAT, alanine aminotransferase; ASAT, aspartate aminotransferase; CRP, C-reactive protein; GORD, gastro-oesophageal reflux disease

### Lower FE levels are associated with older age, longer diabetes duration, smoking and male sex

**Fig. 1:**
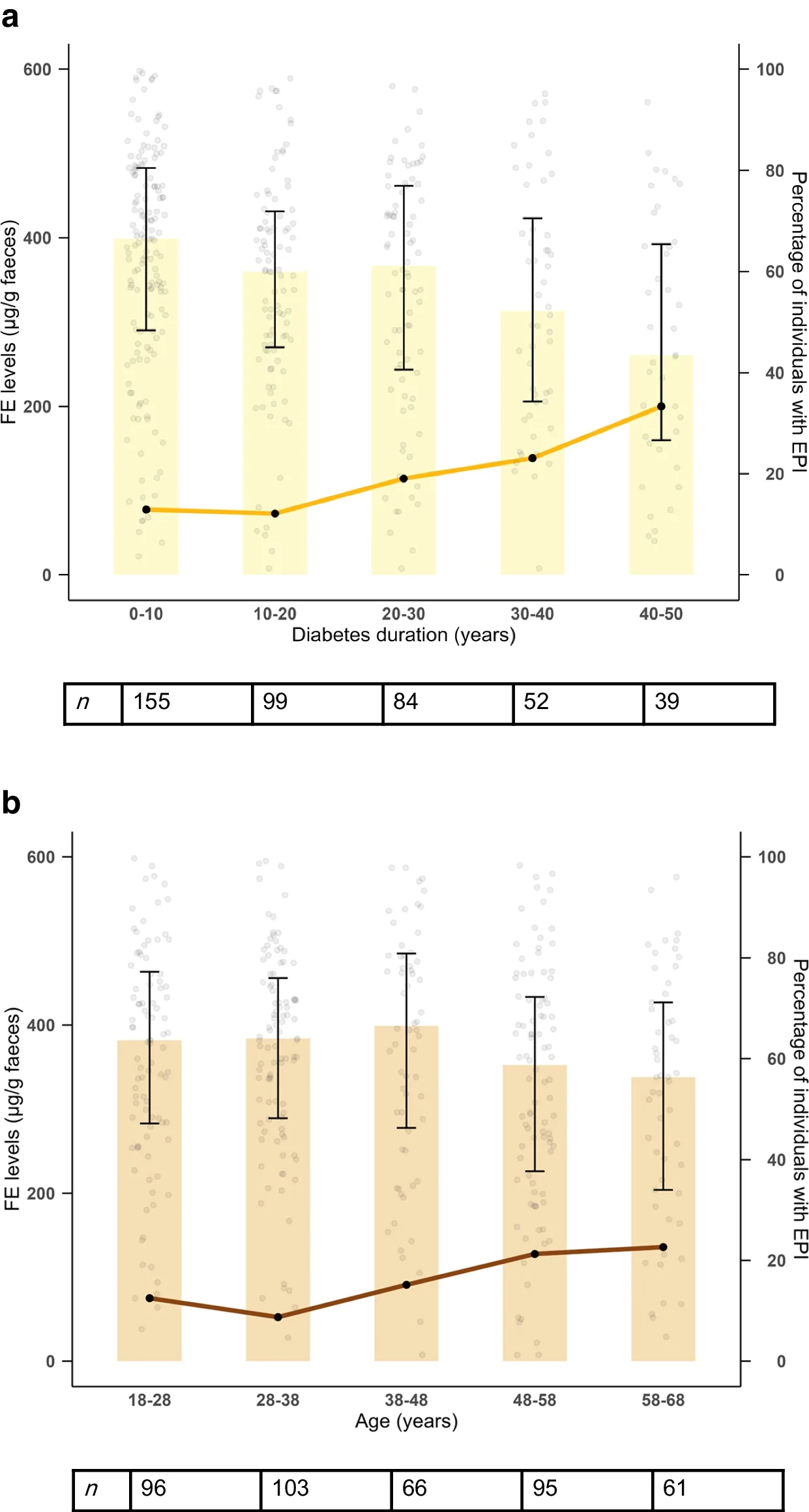
Associations of FE levels with diabetes duration (**a**) and age (**b**). The orange and brown lines represent the percentage of individuals with EPI (defined as FE levels <200 µg/g faeces). Bars represent median and IQR

First, we visualised the relationship between age, duration and FE levels (Fig. 1). Older age and longer diabetes duration were both independently associated with lower FE levels and a higher EPI prevalence. These associations were confirmed using linear regression models (Table 2). Furthermore, smoking and male sex were also independently associated with lower FE levels.

**Table 2:** Results of linear regression models showing the associations of various parameters with FE levels (µg/g faeces)

	Crude			Adjusted		
	B	95% CI	*p* value	B	95% CI	*p* value
Diabetes duration, per 10 years	−21.93	−31.25, −12.61	<0.01	−13.09	−23.54, −2.64	0.01
BMI, per kg/m^2^	−3.84	−7.10, −0.58	0.02	−2.72	−5.84, −0.41	0.09
Smoking status	−94.92	−140.35, −49.48	<0.01	−85.06	−128.51, −41.60	<0.01
Male sex	−64.74	−92.46, −37.02	<0.01	−54.07	−81.41, −26.73	<0.01
Age, per 10 years	−21.50	−30.40, −12.52	<0.01	−10.97	−21.02, −0.92	0.03
Alcohol intake, per unit	−3.20	−6.13, −0.28	0.03	−0.94	−3.77, 1.90	0.52

We used linear regression to estimate B (with 95% CI) per 1 SD (147 µg/g) increase in FE

In the adjusted model, associations were adjusted for all other reported covariates

### Lower FE levels are not associated with altered macronutrient intake

Next, we assessed the association of FE levels with reported macronutrient intakes, testing the hypothesis that lower FE levels may lead to decreased intake due to food avoidance. In unadjusted analyses, lower FE levels were associated with a lower fibre intake, but a significant association was not observed when adjusting for diabetes duration, smoking status, sex, age, alcohol intake and BMI (Table 3).

**Table 3:** Associations between FE levels and macronutrient intake

	Crude			Adjusted		
	Ratio (exp B)	95% CI	*p* value	Ratio (exp B)	95% CI	*p* value
Fat, g/day	1.03	0.99, 1.07	0.20	1.01	0.97, 1.06	0.52
Protein, g/day	1.01	0.98, 1.05	0.42	1.01	0.98, 1.04	0.54
Energy, kJ/day	1.02	0.99, 1.05	0.23	1.01	0.98, 1.04	0.51
Carbohydrate, g/day	1.00	0.96, 1.04	0.98	1.00	0.96, 1.04	0.94
Fibre, g/day	1.05	1.00, 1.09	0.03	1.02	0.98, 1.07	0.38

Ratios (exp B) represent fold changes (with 95% CI) per 1 SD (147 µg/g) increase in FE, using a linear regression model. In the adjusted model, age, sex, diabetes duration, smoking, alcohol use and BMI were added as potential confounders

### Associations between EPI with markers of glycaemic control and residual beta cell function

Next, we tested whether FE levels were associated with markers of glycaemic control (TIR, TBR, TAR, GCV, HbA_1c_, fasting glucose and mean glucose) and residual beta cell function (fasting C-peptide and UCPCR). We found no evidence of association of lower FE levels with markers of residual beta cell function in the adjusted model. Of the glycaemic markers, a lower HbA_1c_ and lower mean glucose were independently associated with higher FE levels in our cohort (Table 4).

**Table 4:** Associations of FE levels with markers of residual beta cell function (fasting C-peptide and UCPCR) and glycaemic management (TIR, TAR, TBR, GCV, HbA_1c_, fasting glucose and mean glucose)

	Crude			Adjusted		
	PR/B/exp (B)^a^	95% CI	*p* value	PR/B/exp (B)^a^	95% CI	*p* value
Detectable C-peptide	1.25	1.08, 1.46	<0.01	1.05	0.92, 1.20	0.46
Detectable UCPCR	1.17	1.06, 1.29	<0.01	1.07	0.98, 1.16	0.12
TIR≥70%	1.14	1.03, 1.26	0.01	1.09	0.97, 1.21	0.14
TAR<26%	1.12	1.00, 1.25	0.04	1.07	0.95, 1.20	0.25
TBR<4%	1.01	0.95, 1.07	0.80	0.98	0.92, 1.04	0.49
GCV<36%	1.11	1.03, 1.21	0.01	1.05	0.97, 1.15	0.22
HbA_1c_, mmol/mol	−1.75	−2.84, −0.66	<0.01	−1.44	−2.59, −0.30	0.01
Fasting glucose, mmol/l	0.97	0.94, 1.01	0.10	0.97	0.93, 1.01	0.11
Mean glucose, mmol/l	0.98	0.96, 0.999	0.04	0.98	0.96, 1.002	0.07

FE levels are expressed per 1 SD increase (147 µg/g faeces). In the adjusted model, age, sex, diabetes duration, smoking, alcohol use and BMI were added as potential confounders

^a^For the dichotomised outcomes, we used Poisson regression with robust standard errors to estimate PR with 95% CI. For HbA_1c_, we used linear regression to estimate B (with 95% CI) per 1 SD (147 µg/g) increase in FE. For fasting glucose and mean glucose, ratios (exp B) represent fold changes (with 95% CI) per 1 SD increase in FE, using a linear regression model

PR, prevalence ratio

### No evidence of association of FE levels with gastrointestinal symptoms, lipid parameters and liver or kidney disease

We also studied markers of potential kidney and liver involvement available in our cohort, as well as abdominal complaints as reported in the GSRS. Higher FE levels were associated with a 6% (95% CI 3–8) lower fibrosis score per SD of FE (Table 5). However, this association was not seen in the adjusted models. Similarly, we observed an association between eGFR in the crude model but not in the adjusted model. We found no evidence of an association between GSRS score and FE levels, either for the total score (Table 5) or the individual components of the GSRS (data not shown). We found a weak association between FE levels and HDL-cholesterol and triglyceride levels, but these associations were attenuated when adjusting for age, sex, BMI, duration, smoking and alcohol use (ESM Table 1).

**Table 5:** Associations of FE levels with markers of kidney and liver damage and GSRS scores

	Crude			Adjusted		
	exp (B)/B^a^	95% CI	*p* value	exp (B)/B^a^	95% CI	*p* value
Liver fibrosis score, kPa^b^	0.94	0.92, 0.97	<0.01	0.97	0.94, 1.00	0.06
GSRS, total score	1.03	1.00, 1.07	0.08	1.01	0.98, 1.05	0.53
eGFR, ml/min^c^	2.19	0.46, 3.92	0.01	−0.20	−1.56, 1.16	0.77

FE levels are expressed per 1 SD increase (147 µg/g faeces). In the adjusted model, age, sex, diabetes duration, smoking, alcohol use and BMI were added as potential confounders

^a^For liver fibrosis score and GSRS, ratios (exp B) represent fold changes (with 95% CI) per 1 SD (147 µg/g) increase in FE, using a linear regression model. For HbA_1c_, we used linear regression to estimate B (with 95% CI) per 1 SD increase in FE

^b^Assessed by liver stiffness measurement using vibration-controlled transient elastography (LSM-VCTE)

^c^Measured using the CKD-EPI method

### EPI status is independently associated with macronutrient intakes and glycaemic management

**Fig. 2:**
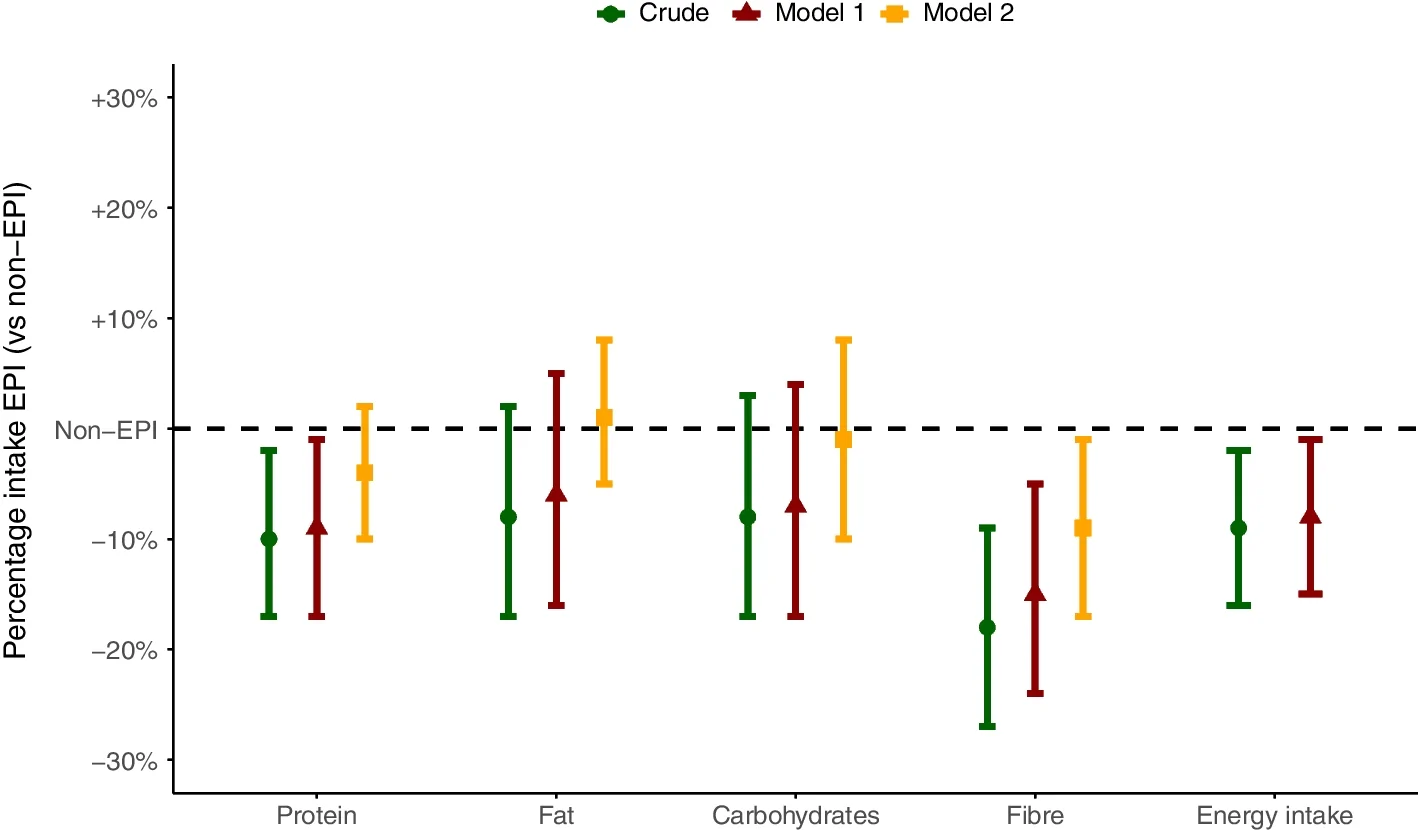
Associations between EPI and macronutrient intake. A linear regression model was used to express the percentage intake of individual macronutrients and total energy intake for those in the EPI group compared with those in the non-EPI group. Model 1 includes age, diabetes duration, smoking status, alcohol intake, sex and BMI as potential confounders. Model 2 includes the confounders for model 1 plus total energy intake. The bars represent 95% CI of the point estimates

Next, we dichotomised FE levels according to EPI status: <200 µg/g faeces (EPI group) or >200 µg/g faeces (non-EPI group), as this cut-off is used in international guidelines [9, 23] and may better capture the non-linear relationship between FE levels and clinical parameters compared with the linear analysis. We confirmed the relationship between FE levels and glycaemic parameters, as EPI was independently associated with a 4.31 mmol/mol (0.39%) higher HbA_1c_ (95% CI 1.35, 7.26) and an 8% higher mean glucose (95% CI 2, 14) (ESM Table 2). However, we did not find any evidence of associations between EPI status and the CGM metrics TIR, TAR, TBR and GCV. Additionally, EPI was independently associated with a 9% lower fibre intake (95% CI 1, 17) and an 8% lower total energy intake (95% CI 1, 15) (Fig. 2 and ESM Table 3). As we have reported an independent association between fibre intake and glycaemic management in a previous study, we hypothesised that fibre intake was confounding the association between EPI and HbA_1c_, but additional adjustment for fibre intake did not change the association (data not shown). For markers of liver and kidney function and abdominal complaints, we found no evidence of associations with EPI in the adjusted models (ESM Table 4). To test the hypothesis that individuals with EPI may be misdiagnosed and may have underlying type 3c diabetes as a result of chronic pancreatitis, we compared the number of individuals with detectable autoantibodies (GADA and IA-2A) between the EPI and non-EPI groups, but found no significant differences (EPI group: 31% of individuals had detectable antibodies; non-EPI group: 27% of individuals had detectable antibodies, *p*=0.60 using the χ^2^ test).

## Discussion

In this cross-sectional cohort of 443 individuals with type 1 diabetes, we assessed the association of FE levels with type 1 diabetes parameters and clinical features. In addition to the known associations of FE levels with disease duration and smoking [10], lower FE levels were associated with a higher HbA_1c_, older age and male sex. We found no evidence of independent associations between FE levels as a continuous variable and other glycaemic markers, liver and kidney function, liver fibrosis score or dietary intake. However, when dichotomising FE levels according to international guidelines, we found an EPI prevalence of 17%, which was associated with a higher HbA_1c_, a higher mean glucose, a lower fibre intake and a lower total energy intake, which has not been described previously. The association with HbA_1c_ and mean glucose is remarkable as we found no evidence of association with CGM metrics, but the ranges for the CGM metrics (e.g. TIR 4–10 mmol/l) may be too broad to detect subtle differences in glycaemia. Taken together, these findings do not justify routine measurement of FE levels in individuals with type 1 diabetes, although clinical awareness of EPI and its complications may be warranted, especially in individuals with longer diabetes duration. Individuals with type 1 diabetes and EPI may be difficult to identify in clinical practice as we found no difference in abdominal complaints between the EPI and non-EPI groups. Previous studies reported that individuals with type 1 diabetes had a lower bone mineral density than healthy control individuals [14], and, in a study of individuals with EPI due to chronic pancreatitis, EPI was associated with a higher risk of osteoporosis (43% vs 6% in those without EPI) and with vitamin deficiencies [24]. These observations, together with our data, raise the possibility that the lower caloric intake observed in individuals with EPI reflects food avoidance due to (subclinical) malabsorption and related symptoms, and these individuals are therefore at risk for complications of EPI.

However, studies involving FE levels, measurements of bone mineral density and plasma vitamin levels in individuals with type 1 diabetes should be conducted to shed further light on this hypothesis. The EPI prevalence in our cohort (17%) is lower than that reported in other cohorts. Indeed, in a cohort of 320 individuals with type 1 diabetes, FE levels <200 µg/g faeces were reported in 51% of participants [10]. Another study in 195 individuals observed FE levels <200 µg/g faeces in 34% of individuals with type 1 diabetes [12]. These studies used a similar measuring technique (polyclonal ELISA), and the diabetes duration was also similar to that in our cohort (median of approximately 15 years). The difference in observed EPI prevalence may be explained by the number of male participants in both populations (64% and 54% vs 37% in our cohort); the number of smokers was not reported in those studies. In an earlier study in the GUTDM1 cohort, we found that higher fibre intake was associated with improved glycaemic management [17]. Remarkably, in the current work, having EPI was found to be associated with both a lower fibre intake and a higher HbA_1c_. Although causality cannot be proven in our study, we speculate that a higher fibre intake attenuates inflammation through the production of short-chain fatty acids, with favourable effects on exocrine function and glycaemic management. To test this hypothesis, the effect of fibre intake on inflammatory parameters and exocrine function should be studied in interventional studies. The fact that several associations observed in the dichotomised analyses were not observed in the linear analyses may indicate a non-linear relationship of FE levels with digestive capacity. In support of a non-linear relationship, the cut-off of 200 µg/g faeces is also recommended by international guidelines [23]. The association of exocrine dysfunction with diabetes duration may be visualised as a gradual decrease, in contrast to the rapid, biphasic loss of C-peptide and proinsulin in type 1 diabetes that we and others have described previously [25, 26]. Our findings imply that clinicians should be aware of the possibility of EPI, especially in older individuals with type 1 diabetes, those who are male and smokers, regardless of the presence of residual beta cell function. Emphasising the entanglement of endocrine and exocrine disease activity in type 1 diabetes, elevated amounts of dendritic cells, CD8-positive T cells [6] and neutrophils [27] have been observed in the exocrine compartment of individuals with type 1 diabetes compared with healthy individuals, and specific antibodies against exocrine enzymes have been described [28]. Pancreatic size is also known to be reduced at the time of type 1 diabetes diagnosis and continues to decline in the years after diagnosis [29]. However, whether exocrine involvement in type 1 diabetes is a consequence of T cell-mediated beta cell destruction or whether exocrine acinar cells are direct targets of autoimmunity remains to be elucidated. The gradual decline in FE levels that we observed, in contrast to the rapid, biphasic decline of endocrine function (as reflected by C-peptide), may hint at separate pathophysiological processes in the exocrine pancreas compared with the endocrine pancreas, but may also simply reflect a larger functional reserve capacity of the exocrine pancreas compared with the endocrine pancreas. We did not observe the classical signs of weight loss and steatorrhea in the EPI group. However, clinically relevant malnutrition may occur before overt clinical symptoms, and steatorrhea only occurs when pancreatic enzyme secretion falls below 10% [30]. In this regard, our finding of a lower caloric intake in the EPI group may suggest undetected EPI, which may be even harder to uncover as individuals with type 1 diabetes have been found to have a tailored, distinct eating pattern compared with healthy control individuals [31]. Given that Whitcomb et al showed that pancreatic enzyme replacement therapy increases protein absorption (determined using a nitrogen absorption coefficient) in individuals with EPI after pancreatectomy [30], individuals with type 1 diabetes and EPI with low protein intake may benefit from pancreatic enzyme replacement therapy. Hence, our findings open the possibility of a trial using pancreatic enzyme replacement therapy in individuals with type 1 diabetes and EPI. The results of such a study could shed light on our hypothesis that the lower caloric intake in our cohort reflects food avoidance due to (subclinical) malabsorption and related symptoms.

### Strengths and limitations

Our cohort is the largest to date to assess EPI prevalence on the basis of FE measurements, and the first to relate EPI in type 1 diabetes to CGM metrics, residual beta cell function and macronutrient intake. However, the current study has several limitations. The cross-sectional design of our study prevents inference of causal relationships. Also, we did not assess other common causes of EPI, such as chronic pancreatitis and cystic fibrosis. However, we believe that the observed EPI prevalence is primarily associated with type 1 diabetes, as the prevalence of EPI in our cohort greatly exceeds the prevalence of these conditions [32, 33]. The high EPI prevalence in other type 1 diabetes cohorts [10, 12] and the absence of an association of EPI with alcohol intake and markers of cholestasis, which are commonly associated with chronic pancreatitis, supports this conclusion. The similar levels of autoantibodies between the EPI and non-EPI groups in our cohort further argue against a higher rate of misdiagnosed individuals with chronic pancreatitis in the EPI group. Furthermore, food intake is known to be under-reported in general [34], and, although we assume that this occurs to a similar extent across the EPI and non-EPI groups, reporting bias cannot be fully excluded, as in most studies using self-reported data. Additionally, we did not directly assess osteoporosis via DEXA scans, which could have shed further light on the relationship between type 1 diabetes and EPI and its complications. Although initially all individuals collected faecal samples, material for elastase measurement was only available in 452 individuals, of which nine were excluded for having self-reported watery stools during sample collection. As seen in ESM Table 5, the excluded group had a higher HbA_1c_, shorter diabetes duration, and, as expected, a higher proportion of self-reported diarrhoea. These exclusions may have introduced bias into the estimated associations, although the direction of this bias is unknown. Age, sex and BMI (as an estimate of adiposity) are well-known factors related to metabolic health, and were therefore included in the models as potential confounders. Diabetes duration is a marker of disease progression and severity, and strongly influences clinical outcomes, and was therefore added as a potential confounder. Alcohol use and smoking are known to be associated with exocrine pancreatic function and are also therefore potential confounders. However, there may be a risk of residual confounding by variables that could not adequately be addressed in our cohort due to its composition and sample size (e.g. ethnicity and family history of diabetes). This should be considered when interpreting the results.

### Conclusion

The lack of evidence of associations of FE levels with clinical parameters in this relatively healthy population of individuals with type 1 diabetes does not support the use of FE measurement for routine screening for EPI. However, the high prevalence of low FE levels and their association with lower food intake and higher glucose levels justify clinical awareness for EPI and its complications. Furthermore, smoking cessation in individuals with type 1 diabetes may not only improve cardiovascular risk but also contribute to preservation of exocrine pancreas function.

## Supplementary Information

Below is the link to the electronic supplementary material.

ESM (PDF 881 KB)

## Data Availability

The data that support the findings of this study are not publicly available due to privacy restrictions. Requests for data access may be submitted via the corresponding author and will be considered on a case-by-case basis for research purposes, subject to institutional approval and relevant ethical and regulatory requirements. Data will be made available only to eligible researchers through the Amsterdam UMC formal application mechanism and may be subject to restrictions on use.

## Abbreviations

CGM: Continuous glucose monitoring
EPI: Exocrine pancreatic insufficiency
FE: Faecal elastase
GCV: Glucose coefficient of variance
GSRS: Gastrointestinal Symptom Rating Scale
TAR: Time above range
TBR: Time below range
TIR: Time in range
UCPCR: Urinary C-peptide to creatinine ratio

## Authors’ relationships and activities

MN is the founder and a scientific advisory board member of Caelus Pharmaceuticals and Advanced Microbiome Interventions (The Netherlands). He is also on the board of directors of Diabeter Netherlands BV. NMJH has received honoraria from Boehringer Ingelheim, Bayer and Novo Nordisk paid to his employer. NMJH has received a research grant from Novo Nordisk. None of these interests are relevant to this article. The remaining authors declare that there are no relationships or activities that might bias, or be perceived to bias, their work.
