# Supplementary material for: High prevalence of low faecal elastase levels in individuals with type 1 diabetes is associated with diabetes duration but not with marked alterations in food intake or clinical symptoms
Source: Diabetologia. 2026 Aug 8;69(11):3221–31. doi: 10.1007/s00125-026-06829-9 (PMC13627185; doi:10.1007/s00125-026-06829-9)
Supplement: Supplementary file 1 [file 125_2026_6829_MOESM1_ESM.pdf]

SUPPLEMENTARY MATERIAL

High prevalence of low faecal elastase levels in individuals with type 1 diabetes is associated with diabetes duration but not with marked alterations in food intake or clinical symptoms

ESM Table 1. Associations of FE levels and plasma lipid levels.

	Crude				Adjusted			
	B	95% CI		*p*-value	B	95% CI		*p*-value
		Lower	Upper			Lower	Upper	
HDL-cholesterol, mmol/l	0.04	0.00	0.08	0.05	0.01	-0.03	0.05	0.59
LDL-cholesterol, mmol/l	-0.01	-0.08	0.06	0.82	0.00	-0.08	0.08	0.97
Triglycerides, mmol/l	-0.04	-0.08	-0.01	0.01	-0.02	-0.05	0.02	0.39
Total cholesterol, mmol/l	0.02	-0.07	0.10	0.68	0.01	-0.08	0.10	0.84

We used linear regression with 95% CI to estimate B per 1 SD (147 µg/g) increase in FE.

In the adjusted model, age, sex, diabetes duration, smoking, alcohol use and BMI are added as potential confounders.

ESM Table 2. Associations of EPI-status (EPI: FE levels <200 μg/g faeces, non-EPI: FE levels ≥200 μg/g faeces) and markers of residual β-cell function (fasting C-peptide and UCPCR) and glycaemic control (TIR, TAR, TBR, GCV, HbA_{1c}, fasting glucose and mean glucose).

	Crude				Adjusted			
	PR/B/exp B[a]	95% CI		*p*-value	PR/B/exp B[a]	95% CI		*p*-value
		Lower	Upper			Lower	Upper	
Detectable C-peptide	1.19	0.80	1.79	0.39	0.88	0.64	1.21	0.43
Detectable UCPCR	1.20	0.91	1.59	0.20	1.03	0.82	1.29	0.79
TIR≥70%	1.40	1.01	1.95	0.04	1.28	0.92	1.77	0.14
TAR<26%	1.41	0.99	1.99	0.06	1.30	0.91	1.84	0.15
TBR<4%	0.95	0.82	1.11	0.50	0.88	0.75	1.03	0.12
GCV<36%	1.37	1.06	1.79	0.02	1.25	0.96	1.63	0.10
HbA_{1c}, mmol/mol	5.28	2.42	8.14	<0.01	4.31	1.35	7.26	<0.01
Fasting glucose, mmol/l	1.07	0.98	1.18	0.14	1.07	0.97	1.18	0.18
Mean glucose, mmol/l	1.08	1.02	1.14	0.01	1.08	1.02	1.14	0.01

[a]For the dichotomised outcomes, we used Poisson regression with robust standard errors to estimate PR with 95% CI. For HbA_{1c}, we used linear regression with 95% CI to estimate B per 1 SD (147 μg/g) increase in FE. For fasting glucose and mean glucose, ratios (exp B) represent fold changes per 1 SD increase in FE, using a linear regression model with 95% CI

In the adjusted model, age, sex, diabetes duration, smoking, alcohol use and BMI are added as potential confounders.

ESM Table 3. Associations of EPI-status and macronutrient intake.

	Ratio (exp B)[a]	95% CI		*p*-value
		Lower	Upper	
	Crude			
Fat, g	0.92	0.83	1.02	0.13
Protein, g	0.90	0.83	0.98	0.02
Energy, kJ	0.91	0.84	0.98	0.01
Carbohydrate, g	0.92	0.83	1.03	0.16
Fibre, g	0.82	0.73	0.91	<0.01
	Model 1			
Fat, g	0.94	0.84	1.05	0.25
Protein, g	0.91	0.83	0.99	0.03
Energy, kJ	0.92	0.85	0.99	0.03
Carbohydrate, g	0.93	0.83	1.04	0.23
Fibre, g	0.85	0.76	0.95	0.01
	Model 2			
Fat, g	1.01	0.95	1.08	0.64
Protein, g	0.96	0.90	1.02	0.17
Carbohydrate, g	0.99	0.90	1.08	0.78
Fibre, g	0.91	0.83	0.99	0.03

[a]Ratios (exp B) represent fold changes per 1 SD increase in FE, using a linear regression model with 95% CI

In the adjusted model, age, sex, diabetes duration, smoking, alcohol use and BMI were added as potential confounders.

ESM Table 4. Associations of EPI-status (EPI: FE levels <200 μg/g faeces, non-EPI: FE levels ≥200 μg/g faeces) and markers of kidney- and liver damage and GSRS-scores.

	Crude				Adjusted			
	Ratio (exp B)/B [a]	95% CI		*p*-value	Ratio (exp B)/B [a]	95% CI		*p*-value
		Lower	Upper			Lower	Upper	
Liver fibrosis score (LSM-VCTE, kPa)[b]	1.11	1.02	1.20	0.01	1.04	0.96	1.12	0.38
GSRS, total score	0.98	0.89	1.07	0.63	1.02	0.92	1.12	0.75
eGFR, ml/min[c]	-7.63	-12.18	-3.08	<0.01	-1.50	-5.02	2.02	0.40

In the adjusted model, age, sex, diabetes duration, smoking, alcohol use and BMI are added as potential confounders.

[a] For liver fibrosis score and GSRS, ratios (exp B) represent fold changes per 1 SD (147 μg/g) increase in FE, using a linear regression model with 95% CI. For HbA_{1c}, we used linear regression with 95% CI to estimate B per 1 SD increase in FE.

[b]Assessed by liver stiffness measurement using vibration-controlled transient elastography (LSM-VCTE)

[c]Measured using the CKD-EPI method

ESM Table 5. Comparison of baseline characteristics of study participants between individuals included and individuals excluded (due to missing samples, n=48, or diarrhoea, n=9).

Characteristic	Included in analysis (*n*=443)	Excluded from analysis (*n*=57)	*p*-value
Female	276±62.3	40±70.2	0.308
Age, years	42.00 (29.00, 54.00)	38.00 (26.00, 49.00)	0.075
BMI, kg/m^2	25.36±4.19	25.33±4.39	0.956
Diabetes duration, years	16.00 (6.00, 29.00)	11.00 (1.00, 24.00)	0.008
Insulin use, U day^{-1}	37.00 (26.92, 52.91)	35.00 (28.00, 45.85)	0.381
Insulin use, U kg^{-1} day^{-1}	0.53±0.29	0.49±0.21	0.292
HbA_{1c}, mmol/l	54.82±11.68	61.65±14.07	<0.001
HbA_{1c}, %	7.17±1.07	7.79±1.29	<0.001
Faecal elastase, µg/g faeces	372.00 (253.00, 461.00)	NA (NA, NA)	NA
Haemoglobin, mmol/l	8.67±0.87	8.35±0.83	0.007
Thrombocytes, 10^9/l	258.94±58.48	278.86±57.01	0.016
Leukocytes, 10^9/l	5.60 (4.60, 6.80)	5.60 (4.90, 7.60)	0.434
eGFR, ml/min/1.73 m^2	100.50±18.69	103.15±22.96	0.329
Alkaline phosphatase, IU/l	77.17±22.17	80.14±29.05	0.389
γ-glutamyl transferase, U/l	15.00 (11.00, 21.00)	14.00 (10.00, 20.25)	0.213
Bilirubin, µmol/l	10.62±5.89	8.57±4.94	0.013
ASAT, IU/l	23.00 (19.00, 28.00)	20.00 (15.50, 25.00)	0.016
ALAT, IU/l	20.00 (16.00, 27.00)	17.00 (13.00, 25.00)	0.018
Fibrosis score, kPa[b]	4.70±1.55	4.49±1.20	0.349
CRP, mg/l	1.20 (0.60, 2.30)	1.40 (0.60, 3.40)	0.097
Fasting C-peptide, nmol/l	0.05 (0.05, 0.08)	0.05 (0.05, 0.18)	0.009
UCPCR	0.00 (0.00, 0.41)	0.04 (0.00, 0.60)	0.286
UCPCR secretors	220±49.7	29±50.9	0.423
Total cholesterol, mmol/l	4.71±0.90	4.63±0.93	0.524
HDL-cholesterol, mmol/l l	1.79±0.45	1.69±0.41	0.113
Non-HDL cholesterol, mmol/l	2.91±0.82	2.94±0.90	0.819
LDL-cholesterol, mmol/l	2.58±0.79	2.56±0.86	0.803
Triglycerides, mmol/l	0.61 (0.45, 0.84)	0.64 (0.48, 1.03)	0.285
Smoking,	43±9.7	7±12.3	0.488
Alcohol intake, units/week	2.00 (0.00, 6.00)	3.00 (0.00, 7.00)	0.284
GADA	1.60 (1.00, 46.70)	1.95 (1.00, 38.32)	0.844
IA-2A	5.00 (5.00, 37.75)	6.00 (5.00, 57.00)	0.516
TIR,%	67.00 (52.00, 80.25)	61.00 (49.00, 77.00)	0.353
TAR,%	29.00 (16.00, 45.00)	35.00 (15.00, 47.00)	0.369

TBR,%	2.00 (1.00, 4.00)	2.00 (0.00, 4.00)	0.243
Glucose variability,%	33.90 (28.60, 39.25)	34.10 (28.92, 40.53)	0.54
Fibre intake, g/day	20.21±9.16	20.54±8.02	0.804
Carbohydrate intake, g/day	160.24±62.05	179.49±64.89	0.038
Fat intake, g/day	74.48±30.93	78.76±30.02	0.35
Protein intake, g/day	69.66±24.20	72.08±20.45	0.493
Caloric intake, kJ/day	6821.34±1972.63	7347.94±2185.85	0.076
Sugar intake, g/day	61.84±31.30	69.57±33.07	0.099
Fat intake, % of total caloric intake	0.41±0.09	0.40±0.07	0.642
GSRS-score, total	25.83 (20.00, 34.08)	29.00 (23.00, 37.00)	0.032
Symptoms			
Diarrhoea	54±12.2	17±29.8	0.001
Abdominal pain	65±14.7	13±22.8	0.399
GORD	31±7.0	7±12.3	0.351
Indigestion	115±26.0	12±21.1	0.162
Constipation	96±21.7	10±17.5	0.123

Values for categorical variables are *n* (%); values for continuous variables are means ± SD or median (Q1, Q3). Values are compared using unpaired t-tests or Mann-Whitney-U test (non-normally distributed data). [a]Measured using the CKD-EPI method. [b]Assessed by liver stiffness measurement using vibration-controlled transient elastography (LSM-VCTE). ALAT, alanine aminotransferase; ASAT, aspartate aminotransferase; CRP, C-reactive protein; GORD, gastro-oesophageal reflux disease.

ESM Fig. 1. PP-plots of adjusted linear regression models used.

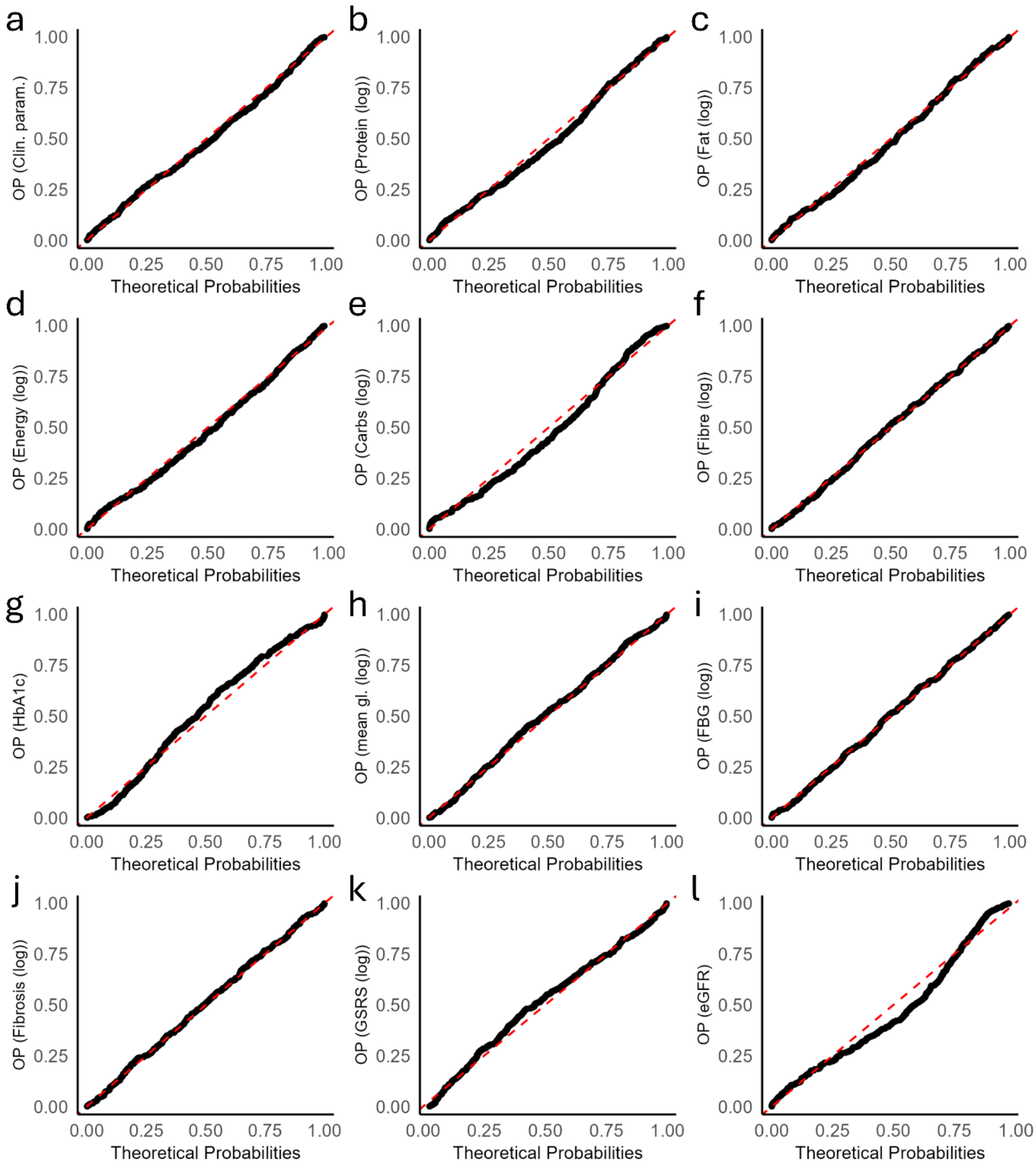

Panel a corresponds to the adjusted model used in Table 2. Panels b-f correspond to the adjusted model used in Table 3. Panels g-i correspond to the adjusted model used in Table 4. Panels j-l correspond to the models used in Table 5. OP, observed probabilities, Clin. param., clinical parameters. Mean gl., mean glucose, FBG, fasting blood glucose.

ESM Fig. 2. QQ-plots of adjusted linear regression models used.

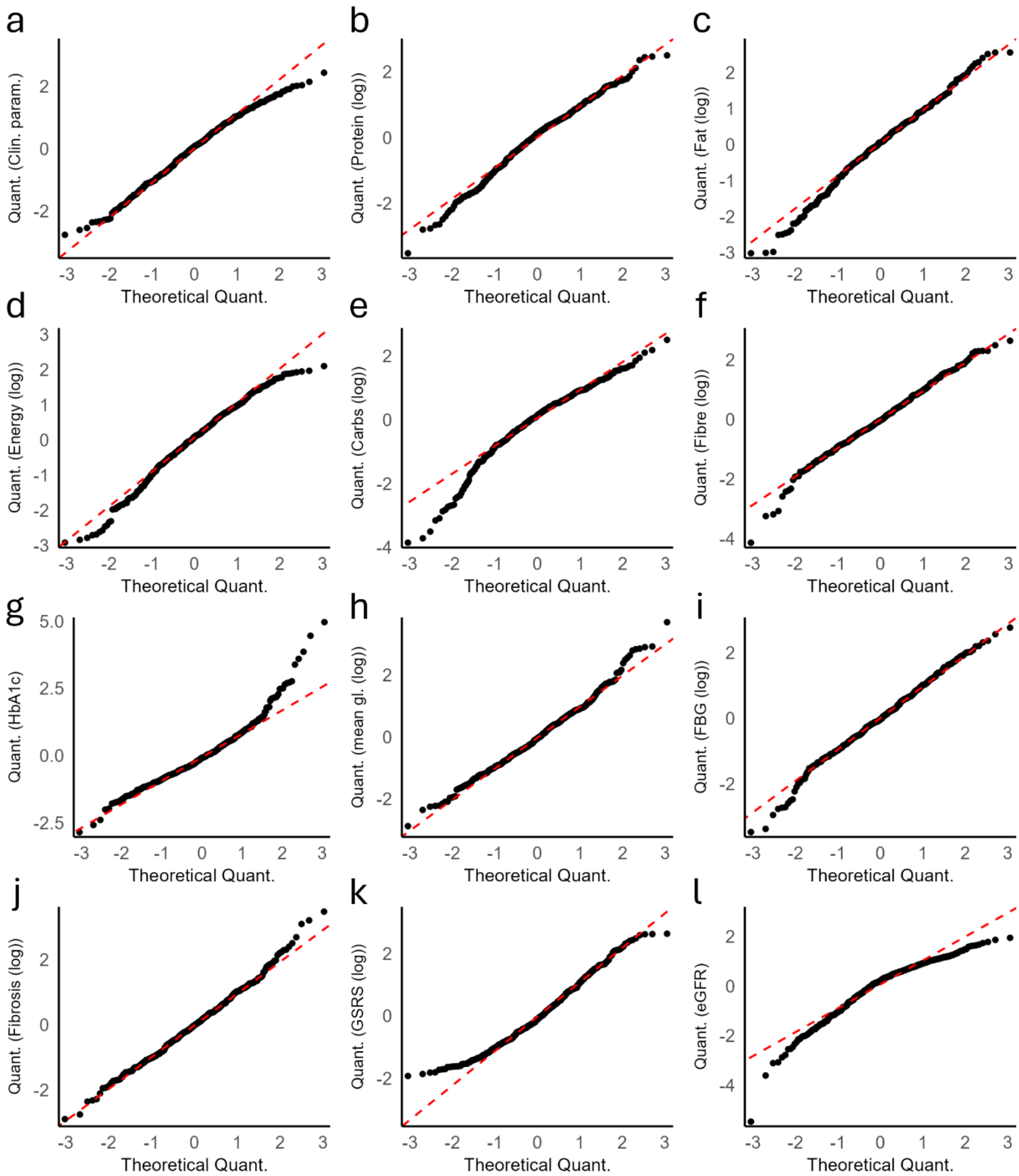

Panel a corresponds to the adjusted model used in Table 2. Panels b-f correspond to the adjusted model used in Table 3. Panels g-i correspond to the adjusted model used in Table 4. Panels j-l correspond to the models used in Table 5. Quant., quantiles, Clin. param., clinical parameters. Mean gl, mean glucose, FBG, fasting blood glucose.
